# Age-Related Declines and Disease-Associated Variation in Immune Cell Telomere Length in a Wild Mammal

**DOI:** 10.1371/journal.pone.0108964

**Published:** 2014-09-30

**Authors:** Christopher Beirne, Richard Delahay, Michelle Hares, Andrew Young

**Affiliations:** 1 Centre for Ecology and Conservation, College of Life and Environmental Sciences, University of Exeter, Penryn Campus, Cornwall, United Kingdom; 2 National Wildlife Management Centre, Animal Health and Veterinary Laboratories Agency, Woodchester Park, Nympsfield, Gloucestershire, United Kingdom; Université de Sherbrooke, Medicine, Canada

## Abstract

Immunosenescence, the deterioration of immune system capability with age, may play a key role in mediating age-related declines in whole-organism performance, but the mechanisms that underpin immunosenescence are poorly understood. Biomedical research on humans and laboratory models has documented age and disease related declines in the telomere lengths of leukocytes (‘immune cells’), stimulating interest their having a potentially general role in the emergence of immunosenescent phenotypes. However, it is unknown whether such observations generalise to the immune cell populations of wild vertebrates living under ecologically realistic conditions. Here we examine longitudinal changes in the mean telomere lengths of immune cells in wild European badgers (*Meles meles*). Our findings provide the first evidence of within-individual age-related declines in immune cell telomere lengths in a wild vertebrate. That the rate of age-related decline in telomere length appears to be steeper within individuals than at the overall population level raises the possibility that individuals with short immune cell telomeres and/or higher rates of immune cell telomere attrition may be selectively lost from this population. We also report evidence suggestive of associations between immune cell telomere length and bovine tuberculosis infection status, with individuals detected at the most advanced stage of infection tending to have shorter immune cell telomeres than disease positive individuals. While male European badgers are larger and show higher rates of annual mortality than females, we found no evidence of a sex difference in either mean telomere length or the average rate of within-individual telomere attrition with age. Our findings lend support to the view that age-related declines in the telomere lengths of immune cells may provide one potentially general mechanism underpinning age-related declines in immunocompetence in natural populations.

## Introduction

Immunosenescence is the gradual deterioration of immune system capability with age, and may be a major factor influencing age-related declines in whole-organism performance [1]. However, the physiological mechanisms that underpin immunosenescence are poorly understood. Biomedical studies have revealed age-related declines in the average length of immune cell telomeres (protective nucleoprotein complexes found at the ends of eukaryotic chromosomes) in humans and laboratory models [2]–[4], and indicate that immune cell telomere lengths can act as biomarkers of age-related disease [5]. This research has led to the suggestion that age-related declines in immune cell telomere lengths may contribute to the immunosenescence-related health declines previously documented in humans and wild vertebrates [6]. Studies of wild birds have now frequently documented age-related declines in the telomere lengths of nucleated erythrocytes (red blood cells) [7], , which share the same haematopoietic stem cell precursors as immune cells. However, despite growing evidence of immunosenescence in wild vertebrate populations [9], it has yet to be investigated whether such age-related declines in telomere length also occur in the immune cell populations of wild vertebrates.

Telomeres consist in part of repetitive sequences of DNA (TTAGGG)^n^ that often decrease in length over time (largely due to oxidative damage and the end replication problem during cell division; [10]) and may trigger cellular senescence once they become critically short [6]. While the enzyme telomerase can recover telomere length, telomerase expression appears to be suppressed in the somatic cells of many mammals, due perhaps to a role for telomere attrition in tumor suppression mechanisms [11]. Age-related declines in the telomere lengths of immune cells *per se* may be expected to arise as (i) many immune cells are constantly renewed via the repeated division of stem cell precursors and (ii) cells of the adaptive immune system (B- and T-lymphocytes) undergo rapid proliferation in response to invading pathogens [12]. As critically short telomeres can compromise the function of haematopoietic stem cells (the cells that produce immune cells and erythrocytes) [11] and reduce the efficacy of immune cell responses (e.g. lymphocyte proliferation potential) [13], age-related declines in immune cell telomere length may act to reduce immunocompetence in older individuals [6].

An important factor influencing immune cell telomere length is disease itself. Observational studies on humans have found shorter immune cell telomere lengths in individuals suffering from age related diseases such as cardiovascular disease and cancer [5]. A comparison of the immune cell telomere lengths of laboratory mice experimentally exposed to the infectious agent *Salmonella enterica* compared to uninfected controls has shown that telomere attrition can also occur as a direct consequence of infectious disease [14]. Despite the clear link between current disease status and immune cell telomere length, to date there have been no assessments of their relationship within a wild vertebrate population naturally infected with disease.

Here we use data from a longitudinal field study of wild European badgers (*Meles meles*) to investigate whether the within-individual age-related declines in immune cell telomere length observed in humans and laboratory models extend to a wild vertebrate population. European badgers are facultatively social and polygynandrous mammals that occupy underground dens (setts). Late life declines in reproductive success consistent with senescence have already been observed in European badgers [15]; however the physiological mechanisms contributing to such declines are currently unknown. As *Mycobacterium bovis* (the causative agent of bovine tuberculosis, bTB), is known to be present in our study population, we also investigate whether shorter immune cell telomeres are associated with disease in the wild.

## Methods

Blood samples were collected from individually-marked badgers of known-age (ranging from 0.3 to 10.3 years) routinely trapped as part of a long-term study at Woodchester Park, Gloucestershire, UK (see [16] for methods). DNA was successfully extracted from 361 buffy coat samples collected from 173 badgers captured and sampled on 1–7 (median = 2) separate occasions between May 2012 and October 2013. Longitudinal measures (individuals captured twice or more) were available for 88 individuals and represented 76% of the total number of observations in the dataset. Average immune cell relative telomere length was determined via a robust and repeatable relative qPCR approach then converted to an absolute telomere length measure (Kb) using standard methods (see Information S1 for complete methodology). The qPCR approach has been successfully utilised to estimate telomere length in numerous ecological studies [7], [17]–[19]. It is particularly well suited to ecological field studies as it requires a small DNA sample, and is cheap and high-throughput in comparison to other methods available [20]. Furthermore, where results from qPCR assays have been compared to other methodologies (such as Terminal Restriction Fragment analysis) they are generally found to be well correlated e.g. [21]. That said, the qPCR technique cannot differentiate ‘true’ telomeric repeats from interstitial telomere-like repeats located away from the chromosome ends [22]. Whilst between-individual differences in the incidence of interstitial repeats have the potential therefore to add noise to comparisons of telomere length between groups of individuals, they are unlikely to influence the results of longitudinal studies (such as this one) where the within-individual change in telomere length is of principal importance [18]. It should also be noted that the absolute estimates of mean immune cell telomere length and telomere attrition rates presented should currently be treated with caution as they have not yet been verified with a second independent methodology [20], [23]. That said, their inclusion here at least offers the potential for future comparisons with other studies employing a similar methodology [24]. All work was approved by the Food and Environment Research Agency Ethical Review Committee and carried out under licence granted by the Home Office under the 1986 Animal (Scientific Procedures) Act.

### Infection Status

Current bTB infection status was assessed at each capture event using three diagnostic tests: i) the interferon-gamma (IFNγ) test, an enzyme immunoassay assessing lymphocyte responsiveness to an *M. bovis* purified protein derivative, ii) the STAT-PAK (Chembio Diagnostic Systems, Inc) test, a lateral-flow immunoassay to identify the presence of *M. bovis* antibodies, and iii) microbiological culture of clinical samples (i.e. sputum, faeces, urine and wound/abscess swabs) to isolate *M. bovis* (see [25], [26] for discussion of the performance of each test). We used a simplified version of the disease classification discussed in detail in [16]. Briefly, individuals were classed as ‘negative’ if they had never tested positive for bTB on any test, ‘positive’ if they had ever tested positive using the STAT-PAK or IFNγ tests, or ‘excretor’ if *M. bovis* was isolated by culture. Thirteen individuals transitioned to a more advanced disease statuses during the course of the study: eleven individuals transitioned from disease ‘negative’ to ‘positive’, one individual from ‘positive’ to ‘excretor’ and one individual from ‘negative’ to ‘excretor’. None of the individuals in this study have ever been vaccinated against bTB.

### Seasonality-corrected body condition

In order to control for variation in body condition (which could conceivably be correlated with age or disease status) in the telomere length analysis [27], we calculated a metric for body condition that accounted for the marked seasonal variation in body mass exhibited by European badgers. To do so, we first calculated the Scaled Mass Index (SMI) for all individuals at each capture, following [28]. This approach factors out variation in body mass arising from variation in body size (in this case utilising body length (cm) as the metric of body size), by scaling the body mass measure for each capture to that for an individual of average size (in this case, of body length = 80 cm). The SMI scaling factor was estimated to be 3.63. We then corrected all SMI measures for seasonal variation by taking residuals from a model of SMI that controlled for variation arising from the month of the year on which the individual was captured.

### Statistical analyses

Competing hypotheses for the causes of variation in telomere length were compared using multi-model inference [29] with linear mixed-models. *A priori* candidate models (n = 28) were defined containing additive effects of all candidate explanatory variables: partitioned age (see explanation below), sex, bTB status, seasonally-corrected body condition and all biologically relevant two way interactions (see Information S2 for full model selection table). A ‘top model set’ was defined, which included all models with ΔAICc≤6 from the best supported model, after excluding any models of which a simpler nested version attained stronger support (following the ‘nesting rule’ of [30]). All co-efficients were model-averaged across the models in the ‘top model set’ in which they occurred using the MuMIn package [31]. Model-averaged coefficients (effects sizes) are discussed in terms of their relative ‘weights’. In order to ensure that our statistical assessment of the within-individual change in telomere length with age was not confounded by between-individual effects (e.g. selective disappearance), we applied a within-subject centring approach, following [32]. Age was partitioned into (i) an individual's ‘mean age’ across all samples collected for that individual, and (ii) its ‘Δ age’ (the offset of its age at the focal sampling point from its mean age, the effect of which reflects within-individual changes in telomere length with age). Previous longitudinal and cross-sectional studies of immune cell telomere lengths have suggested that the rate of telomere attrition may slow with increasing age [33], [34]. To address this possibility, we assess the strength of evidence for quadratic effects of each partitioned age parameter and for an interaction between a mean age and Δ age. To account for repeated measures, qPCR plate heterogeneity, and variation in territory quality, we included ‘individual ID’ nested within ‘plate ID’ (all samples from the same individual were run on the same plate) and ‘social group’ as random intercept terms. Goodness-of-fit was assessed through calculating conditional (total variance explained by the best supported model) and marginal (variance explained by fixed effects alone) R^2^ formulations [35] and standard residual plot techniques.

## Results

**Figure 1:**
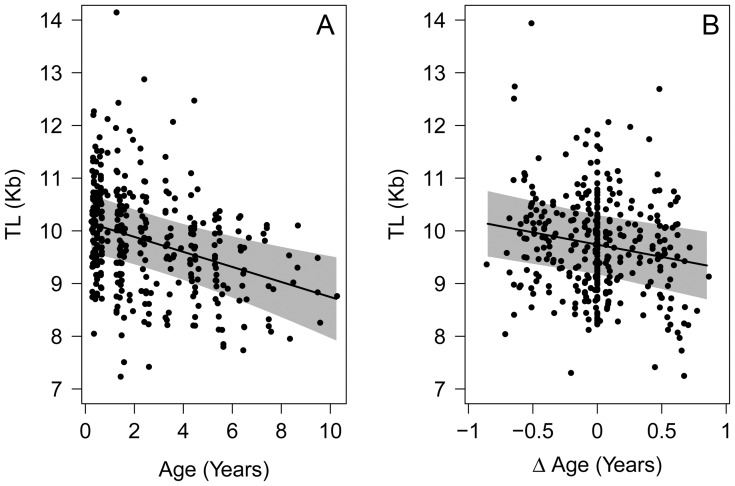
How telomere length changes with age and partitioned age. (A) Relationship between immune cell telomere length (TL) and age (years) whilst controlling for random effects; (B) and (C) Relationship between TL and Δ age (within-individual changes in age) and TL and mean age (between-individual changes in age) respectively while controlling for random effects, bTB infection status, and between-individual differences in age (Table 1); Black lines present model averaged predictions, shaded areas present 95% confidence intervals, and black points present residuals from the best supported models.

The relationship between age and immune cell telomere length, after accounting for the effects of the random factors, is strongly suggestive of the predicted age-related decline (Figure 1A). Crucially, partitioning variance in age into within- and between-individual variation yielded full statistical support for age-related declines in telomere length occurring within individuals (Figure 1B and Table 1). The rate of telomere attrition with age occurring *within* individuals (mean±SE = 460±170 bp/year) was estimated to be over three times faster than that for *between*-individual increases in age (mean±SE = 140±40 bp/year; Table 1). We found no statistical support for quadratic age effects or an interaction between mean age and Δ age, suggesting that the rate of change in mean immune cell telomere length does not change with age.

**Table 1:** Model averaged output of linear mixed model analysis of the factors affecting telomere length: Σ = model weight, SE = effect size standard error, CI = confidence interval, ^a^ = factor levels.

Parameter	Σ	Effect size	SE	95% CI
Mean Age (Years)	1.0	−0.14	0.04	(−0.22 to −0.05)
Delta Age (Years)	1.0	−0.46	0.17	(−0.80 to −0.12)
Disease	0.7	−	-	-
- ^a^Negative		0.00	-	-
- ^a^Positive		0.24	0.23	(−0.03 to 0.74)
- ^a^Excretor		−0.26	0.37	(−1.13 to 0.40)
Sex	0.0	-	-	-
Condition	0.0	-	-	-

Interaction terms without support are not shown. For full model output see Information S2.

**Figure 2:**
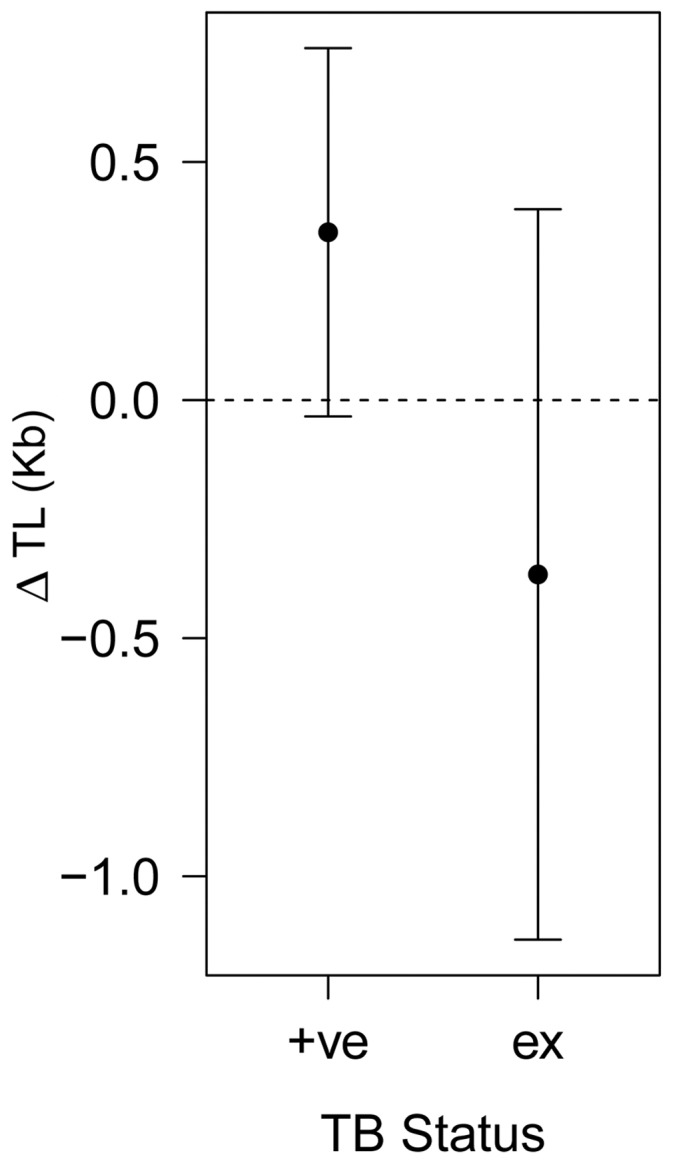
Predicted change in TL due to bTB infection status. Where the dashed line is the predicted TL of bTB negative individuals, ‘+ve’ denotes disease positive individuals, and ‘ex’ individuals classed as excretors. The points present model averaged predictions and error bars present their 95% confidence intervals.

We also found some support for bTB infection status predicting telomere length (Table 1), whereby bTB positive individuals had longer telomere lengths than negative individuals, while individuals in advanced stages of infection (‘excretors’) showed shorter telomere lengths than badgers that tested positive (Figure 2). No support was found for bTB infections status influencing the rate of within-individual telomere attrition (an interaction between bTB status and Δ age). Likewise, no support was found for sex or current body condition influencing telomere length. The proportion of variance explained by the best performing model was 59.7%, though the fixed effects alone (age and disease) accounted for just 5.1% of the total variance.

## Discussion

Our findings strongly suggest that the age-related declines in immune cell telomere length observed in humans and laboratory models are mirrored by comparable relationships in this population of wild mammals. These results therefore lend strength to the suggestion that age-related declines in immune cell telomere length may contribute to the emergence of the immunosenescent phenotypes increasingly documented in wild vertebrate populations [9]. Our findings are also suggestive of associations between immune cell telomere lengths and disease status in the wild, echoing the findings of biomedical research in which human immune cell telomere lengths have been identified as a risk factor for disease [5]. Below we discuss the causal mechanisms that may underpin the relationships observed and their implications, in light of existing work on the telomere dynamics of other cell types in wild vertebrates, for our understanding of patterns of senescence in the wild.

Our findings suggest that the immune cell populations of wild European badgers experience a decline in mean telomere length of approximately 460 bp/year with increasing organismal age. This rate of decline falls between recent estimates for the immune cell telomere attrition rates of humans (72 bp/year) and laboratory mice (7000 bp/year) [2] and is consistent with the previously documented negative correlation between telomere attrition rate and lifespan [4]. The observed age-related declines in mean immune cell telomere length could principally reflect age-related changes in the telomere lengths of particular immune cell classes, the immunological implications of which will depend upon the cell class(es) involved. For example, the observed patterns may principally reflect age-related declines in the telomere lengths of neutrophils as they typically comprise around 80% of total immune cell counts in the European badger [36], [37]. As the high turnover rate of neutrophils is thought to leave their telomere lengths reflecting those of the haematopoietic stem cells from which they derive [12], [38], it seems likely that our results reflect, at least in part, age-related declines in haematopoietic stem cell telomere length. To the extent that this is the case, our findings echo those of studies of wild birds, in which the telomere lengths of nucleated erythrocytes (which also derive from the haematopoietic stem cells) have typically been found to decrease with age [4], [7], [8]. Collectively these findings highlight the possibility that pathological consequences of short telomeres in this stem cell compartment [11],[39] may contribute to age-related declines in whole-organism performance. However, as lymphocytes also represent a significant proportion of European badger immune cells (∼20%; [36], [37]), our findings may also reflect, at least in part, age-related declines in lymphocyte telomere lengths, whose dynamics may differ markedly from those of the haematopoietic stem cells. Age-related declines in the telomere lengths of lymphocytes *per se* have previously been documented in humans and laboratory models [33], [40], and, given the potential for short telomeres to limit lymphocyte proliferation capacity [13], have been implicated as one potential driver of late-life declines in the capability of the adaptive immune system. The application of cell sorting to field-derived samples of immune cell populations prior to telomere analysis might now be usefully prioritised, to clarify which immune cell sub-types are exhibiting age-related declines in telomere length and, by extension, the immunological implications of such declines.

While our findings could indeed reflect age-related declines in the telomere lengths of specific immune cell subsets that might thereby contribute to the emergence of immunosenescent phenotypes, there are several reasons for caution when considering this possibility. First, it is also possible that the observed age-related changes in immune cell mean telomere length instead arise in part from changes with age in the ratios of immune cell sub-types in circulation [41], which may differ in mean telomere length (e.g. an increasing representation of memory T-cells with shorter telomeres relative to naïve T-cells with longer telomeres) [6]. Ultimately, the relative contributions of telomere attrition *per se* versus age-related changes in immune cell composition will only become clear once cell-type-specific telomere length estimates can be coupled with knowledge of age-related changes in the ratios of these same cell types, which is currently an ambitious prospect for studies of non-model organisms. Second, even if the observed age-related changes do arise from within-cell-type changes in telomere length, our statistical models highlight that the fixed effects alone (age and disease status) explain only a small proportion of the observed variation in mean immune cell telomere length (just 5%), suggesting that the biological significance of these within-individual changes with age may be limited relative, for example, to the processes that generate between-individual variation. Finally, while the observed age-related declines in mean telomere length could be a pathological consequence of cumulative exposure to the diverse processes that may shorten immune cell telomere lengths (e.g. oxidative stress or disease), it is also possible that they reflect, in part or whole, adaptive changes in immunological investment with age (either in telomere maintenance or immune cell sub-type ratios), given, for example, a reduced likelihood that older individuals will encounter novel pathogens. Whether naturally occurring age-related changes in immune cell telomere length necessarily would contribute to late-life declines in whole organism performance in natural populations under ecologically realistic conditions therefore remains open to debate.

Our findings are also suggestive of an association between disease state and immune cell telomere length in a natural vertebrate population under ecologically realistic conditions, again echoing studies of humans and laboratory models [14] However, it must be noted that due to the low frequency of indviduals transitiong between disease states within the study period these patterns are largely cross-sectional in nature. The initial increase in telomere length with the transition to testing positive for bTB was unexpected; however, previous work in humans found telomerase activity to be present in immune cells of individuals with pulmonary tuberculosis and absent in healthy controls [42]. As the immune responses to bTB comprise of both cell-mediated and humoral components [43], involving T and B-cells which are known to express telomerase [6], [44], telomerase expression in immune cell subsets during infection may be contributing to this pattern. The apparent decrease in immune cell telomere length associated with progression from testing positive for bTB to excreting the bacterium is consistent with evidence that disease progression in humans is linked to the shortening of immune cell telomeres [3]. Whether this association reflects short telomeres pre-disposing individuals to disease progression [6], the impacts of infection on immune cell telomere length [14], or indeed a role for other processes that may both shorten immune cell telomeres and leave individuals pre-disposed to disease progression, remains unclear. It is also conceivable that these patterns reflect disease-associated variation in the composition of the immune cell population from which our telomere length assessments derive, rather than variation in the telomere lengths of specific cell types. As the presence of critically short telomeres (rather than an overall decline in population average telomere length) may act as the crucial factor linking disease progression and immune cell viability [45], [46], this possible link between disease status and telomere dynamics may be more appropriately addressed using methodologies with single chromosome resolution [see 20].

Our findings have implications too for a growing body of work seeking to understand the causes and consequences of sex differences in telomere dynamics [47]. It has been argued, for example, that males might be expected to show shorter telomeres and/or experience higher rates of telomere attrition in species that exhibit male-biased sexual size dimorphism, male-biased mortality rates and/or steeper rates of senescence decline among males than females (reviewed in [47]). As all three phenomena have been documented in the European badger [15], [48], [49], it is notable that our findings reveal no evidence of a sex difference in either mean immune cell telomere length or in the within-individual rate of immune cell telomere attrition with age. Our findings therefore echo recent work highlighting the likely complexity of the proximate and ultimate causes of sex differences in telomere dynamics, and the difficulty of drawing generalisations from the small number of studies in this area to date [47].

In summary, our findings provide the first evidence, to our knowledge, of within-individual declines in immune cell telomere length with age in a wild vertebrate, suggesting that the immune cell telomere attrition documented in humans and laboratory models may indeed generalise to natural vertebrate populations experiencing ecologically realistic conditions. As such, while their immunological implications will only become clear with further research, our findings do lend strength to the view that age-related declines in immune cell telomere length may offer one potentially general mechanism contributing to declines in both immune and whole-organism performance with age. It remains to be tested whether short immune cell telomeres are associated with weaker whole-organism performance in the wild as appears to be the case for erythrocyte telomere length in wild birds [7], [50], [51]. However, that our statistical models estimate the rate of within-individual age-related decline in immune cell telomere length to be roughly three times faster than the rate of between-individual decline is at least consistent with a scenario where individuals with shorter immune cell telomeres and/or faster telomere attrition rates are selectively disappearing from this population. A key challenge now will be establishing the causes of variation among individuals in the rate of immune cell telomere attrition with age, and the extent to which such variation has causal effects on late-life health and performance.

## Supporting Information

Data S1 **Data used in the analyses of “Age-related declines and disease-associated variation in immune cell telomere length in a wild mammal.”**
(XLSX)

Information S1 **Detailed protocols for the relative and absolute qPCR methodologies.**
(DOCX)

Information S2 **Full model selection table output from data analysis.** Where: numbers denote β-estimates of continuous variables and ‘X’ denotes categorical variables included in each given model (row), INT = Intercept, MA = Mean Age (Years), DA = Delta Age (Years), SMI = Scaled Mass Index, TB = bTB infection status, : = interactions terms, df = degrees of freedom, LogLik = Log Likelihood, Δ = change in AICc from best model, W = Model Weight, AW = Strength of support for top model set (Δ AIC<6 from the best supported model and removal of more complex models with less support than a simpler nested version [9]), and bold typeface denotes the models in the top model set.
(DOCX)

## References

[1] Larbi A, Franceschi C, Mazzatti D, Solana R, Wikby A, et al (2008) Aging of the immune system as a prognostic factor for human longevity. Physiology 23: 64–74. 18400689 10.1152/physiol.00040.2007

[2] Vera E, Bernardes de Jesus B, Foronda M, Flores JM, Blasco MA (2012) The rate of increase of short telomeres predicts longevity in mammals. Cell Rep 2: 1–6. 23022483 10.1016/j.celrep.2012.08.023

[3] Armanios M (2013) Telomeres and age-related disease: how telomere biology informs clinical paradigms. J Clin Invest 123: 996–1002. 23454763 10.1172/JCI66370 PMC3673231

[4] Haussmann MF, Winkler DW, O'Reilly KM, Huntington CE, Nisbet ICT, et al (2003) Telomeres shorten more slowly in long-lived birds and mammals than in short-lived ones. Proc Biol Sci 270: 1387–1392. 12965030 10.1098/rspb.2003.2385 PMC1691385

[5] Fossel M (2012) Use of Telomere Length as a Biomarker for Aging and Age-Related Disease. Curr Transl Geriatr Exp Gerontol Rep 1: 121–127.

[6] Weng N (2012) Telomeres and immune competency. Curr Opin Immunol 24: 470–475. 22626625 10.1016/j.coi.2012.05.001 PMC3423542

[7] Barrett E, Burke T, Hammers M, Komdeur J, Richardson D (2012) Telomere length and dynamics predict mortality in a wild longitudinal study. Mol Ecol 22: 249–259. 23167566 10.1111/mec.12110

[8] Salomons HM, Mulder GA, van de Zande L, Haussmann MF, Linskens MHK, et al (2009) Telomere shortening and survival in free-living corvids. Proc Biol Sci 276: 3157–3165. 19520803 10.1098/rspb.2009.0517 PMC2817122

[9] Palacios MG, Winkler DW, Klasing KC, Hasselquist D, Vleck CM (2011) Consequences of immune system aging in nature: a study of immunosenescence costs in free-living Tree Swallows. Ecology 92: 952–966. 21661557 10.1890/10-0662.1

[10] Monaghan P, Haussmann MF (2006) Do telomere dynamics link lifestyle and lifespan? Trends Ecol Evol 21: 47–53. 16701469 10.1016/j.tree.2005.11.007

[11] Flores I, Benetti R, Blasco M (2006) Telomerase regulation and stem cell behaviour. Curr Opin Cell Biol 18: 254–260. 16617011 10.1016/j.ceb.2006.03.003

[12] Goronzy JJ, Fujii H, Weyand CM (2006) Telomeres, immune aging and autoimmunity. Exp Gerontol 41: 246–251. 16427234 10.1016/j.exger.2005.12.002

[13] Effros R (2011) Telomere/telomerase dynamics within the human immune system: effect of chronic infection and stress. Exp Gerontol 46: 135–140. 20833238 10.1016/j.exger.2010.08.027 PMC3246363

[14] Ilmonen P, Kotrschal A, Penn DJ (2008) Telomere attrition due to infection. PLoS One 3: e2143. 18478110 10.1371/journal.pone.0002143 PMC2366059

[15] Dugdale HL, Pope LC, Newman C, Macdonald DW, Burke T (2011) Age-specific breeding success in a wild mammalian population: selection, constraint, restraint and senescence. Mol Ecol 20: 3261–3274. 21714821 10.1111/j.1365-294X.2011.05167.x

[16] Delahay R, Walker N, Smith G, Wilkinson D, Clifton-Hadley R, et al (2013) Long-term temporal trends and estimated transmission rates for Mycobacterium bovis infection in an undisturbed high-density badger (Meles meles) population. Epidemiol Infect 141: 1445–1456. 23537573 10.1017/S0950268813000721 PMC9151602

[17] Heidinger BJ, Blount JD, Boner W, Griffiths K, Metcalfe NB, et al (2012) Telomere length in early life predicts lifespan. Proc Natl Acad Sci 109: 1743–1748. 22232671 10.1073/pnas.1113306109 PMC3277142

[18] Nettle D, Monaghan P, Boner W, Gillespie R, Bateson M (2013) Bottom of the Heap: Having Heavier Competitors Accelerates Early-Life Telomere Loss in the European Starling, Sturnus vulgaris. PLoS One 8: e83617. 24386235 10.1371/journal.pone.0083617 PMC3873947

[19] Stier A, Viblanc VA, Massemin-Challet S, Handrich Y, Zahn S, et al (2013) Starting with a handicap: phenotypic differences between early- and late-born king penguin chicks and their survival correlates. Funct Ecol 28: 601–611.

[20] Nussey DH, Baird D, Barrett E, Boner W, Fairlie J, et al (2014) Measuring telomere length and telomere dynamics in evolutionary biology and ecology. Methods Ecol Evol 5: 299–310. 25834722 10.1111/2041-210X.12161 PMC4375921

[21] Aviv A, Hunt SC, Lin J, Cao X, Kimura M, et al (2011) Impartial comparative analysis of measurement of leukocyte telomere length/DNA content by Southern blots and qPCR. Nucleic Acids Res 39: e134. 21824912 10.1093/nar/gkr634 PMC3203599

[22] Foote CG, Vleck D, Vleck CM (2013) Extent and variability of interstitial telomeric sequences and their effects on estimates of telomere length. Mol Ecol Resour 13: 417–428. 23398661 10.1111/1755-0998.12079

[23] Horn T, Robertson BC, Gemmell NJ (2010) The use of telomere length in ecology and evolutionary biology. Heredity 105: 497–506. 20736972 10.1038/hdy.2010.113

[24] Barrett ELB, Boner W, Mulder E, Monaghan P, Verhulst S, et al (2012) Absolute standards as a useful addition to the avian quantitative PCR telomere assay. J Avian Biol 43: 571–576.

[25] Chambers M, Waterhouse S, Lyashchenko K, Delahay R, Sayers R, et al (2009) Performance of TB immunodiagnostic tests in Eurasian badgers (Meles meles) of different ages and the influence of duration of infection on serological sensitivity. BMC Vet Res 5: 42. 19919697 10.1186/1746-6148-5-42 PMC2784444

[26] Chambers Ma, Pressling Wa, Cheeseman CL, Clifton-Hadley RS, Hewinson RG (2002) Value of existing serological tests for identifying badgers that shed Mycobacterium bovis. Vet Microbiol 86: 183–189. 11900953 10.1016/s0378-1135(02)00012-3

[27] Caprioli M, Romano M, Romano A, Rubolini D, Motta R, et al (2013) Nestling telomere length does not predict longevity, but covaries with adult body size in wild barn swallows. Biol Lett 9: 20130340. 23883575 10.1098/rsbl.2013.0340 PMC3971670

[28] Peig J, Green AJ (2010) The paradigm of body condition: a critical reappraisal of current methods based on mass and length. Funct Ecol 24: 1323–1332.

[29] Burnham K, Anderson D (2002) Model selection and multi-model inference: a practical information-theoretic approach. Springer

[30] Richards S, Whittingham M, Stephens P (2011) Model selection and model averaging in behavioural ecology: the utility of the IT-AIC framework. Behav Ecol Sociobiol 65: 77–89.

[31] Barton K (2014) Package “MuMIn.” Available: http://cran.r-project.org/web/packages/MuMIn/MuMIn.pdf.

[32] Van de Pol M, Wright J (2009) A simple method for distinguishing within- versus between-subject effects using mixed models. Anim Behav 77: 753–758.

[33] Baerlocher GM, Mak J, Roth A, Rice KS, Lansdorp PM, et al (2003) Telomere shortening in leukocyte subpopulations from baboons. J Leukoc Biol 73: 289–296. 12554806 10.1189/jlb.0702361

[34] Aubert G, Baerlocher GM, Vulto I, Poon SS, Lansdorp PM (2012) Collapse of telomere homeostasis in hematopoietic cells caused by heterozygous mutations in telomerase genes. PLoS Genet 8: e1002696. 22661914 10.1371/journal.pgen.1002696 PMC3355073

[35] Nakagawa S, Schielzeth H (2013) A general and simple method for obtaining R2 from generalized linear mixed-effects models. Methods Ecol Evol 4: 133–142.

[36] McLaren G, Macdonald D, Georgiou C, Mathews F, Newman C, et al (2003) Leukocyte coping capacity: a novel technique for measuring the stress response in vertebrates. Exp Physiol 88: 541–546. 12861342 10.1113/eph8802571

[37] Tomlinson AJ (2013) Life-history correlates of Myobacterium bovis infection in individual Eurasion badgers (Meles meles) University of Liverpool. Available: http://ethos.bl.uk/Home.do.

[38] Aubert G, Lansdorp P (2008) Telomeres and aging. Physiol Rev 88: 557–579. 18391173 10.1152/physrev.00026.2007

[39] Blasco MA (2007) Telomere length, stem cells and aging. Nat Chem Biol 3: 640–649. 17876321 10.1038/nchembio.2007.38

[40] Alter BP, Rosenberg PS, Giri N, Baerlocher GM, Lansdorp PM, et al (2012) Telomere length is associated with disease severity and declines with age in dyskeratosis congenita. Haematologica 97: 353–359. 22058220 10.3324/haematol.2011.055269 PMC3291588

[41] Nussey DH, Watt K, Pilkington JG, Zamoyska R, McNeilly TN (2012) Age-related variation in immunity in a wild mammal population. Aging Cell 11: 178–180. 22107028 10.1111/j.1474-9726.2011.00771.x PMC3397677

[42] Yang C, Lin M, Huang C, Chen N, Chen J (1999) Tuberculin purified protein derivative up-regulates the telomerase activity of peripheral blood mononuclear cells from patients with pulmonary tuberculosis. Life Sci 61: 1383–1391. 10.1016/s0024-3205(99)00072-7 10321718

[43] Flynn J, Chan J (2001) Immunology of tuberculosis. Annu Rev Immunol 19: 93–129. 11244032 10.1146/annurev.immunol.19.1.93

[44] Weng NP, Granger L, Hodes RJ (1997) Telomere lengthening and telomerase activation during human B cell differentiation. Proc Natl Acad Sci 94: 10827–10832. 9380719 10.1073/pnas.94.20.10827 PMC23499

[45] Hemann MT, Strong MA, Hao LY, Greider CW (2001) The shortest telomere, not average telomere length, is critical for cell viability and chromosome stability. Cell 107: 67–77. 11595186 10.1016/s0092-8674(01)00504-9

[46] Lin TT, Norris K, Heppel NH, Pratt G, Allan JM, et al (2014) Telomere dysfunction accurately predicts clinical outcome in chronic lymphocytic leukaemia, even in patients with early stage disease. Br J Haematol 1–10. 10.1111/bjh.13023 24990087

[47] Barrett ELB, Richardson DS (2011) Sex differences in telomeres and lifespan. Aging Cell 44: 913–921. 10.1111/j.1474-9726.2011.00741.x 21902801

[48] Graham J, Smith GC, Delahay RJ, Bailey T, McDonald Ra, et al (2013) Multi-state modelling reveals sex-dependent transmission, progression and severity of tuberculosis in wild badgers. Epidemiol Infect 1–8. 10.1017/S0950268812003019 PMC9151592 23290694

[49] Johnson DDP, Macdonald DW (2001) Why are group-living badgers (Meles meles) sexually dimorphic? J Zool 255: 199–204.

[50] Angelier F, Vleck CM, Holberton RL, Marra PP (2013) Telomere length, non-breeding habitat and return rate in male American redstarts. Funct Ecol 27: 342–350.

[51] Geiger S, Le Vaillant M, Lebard T, Reichert S, Stier A, et al (2011) Catching-up but telomere loss: half-opening the black box of growth and ageing trade-off in wild king penguin chicks. Mol Ecol 21: 1500–1510. 22117889 10.1111/j.1365-294X.2011.05331.x

