# Supplementary material for: Age-Related Declines and Disease-Associated Variation in Immune Cell Telomere Length in a Wild Mammal
Source: PLoS One. 2014 Sep 30;9(9):e108964. doi: 10.1371/journal.pone.0108964 (PMC4182606; doi:10.1371/journal.pone.0108964)
Supplement: Information S1 — Detailed protocols for the relative and absolute qPCR methodologies. (DOCX) [file pone.0108964.s002.docx]

**Information S1**

**Full qPCR protocol**

*Sample acquisition*

A 4ml heparinised blood sample was obtained by venipuncture from each captured badger and spun at 3000 rpm for 10 minutes within 30 minutes of the sample being taken. A 40µl aliquot of the resultant buffy coat (immune cell layer) was stored at -80°C until DNA extraction. Buffy coat samples were gently thawed on ice then DNA was extracted using Fermentas Whole Blood DNA extraction spin columns according to the manufacturer’s protocol. DNA was eluted in 100μl of low EDTA TE buffer and stored at -20°C until qPCR analysis. Average DNA yield (ng/ul) was 57.1 (SD±29) and average DNA purity (A_260_/A_280_) was 1.83 (SD±0.16). DNA integrity was validated by electrophoresis on a 0.7% agarose gel. No evidence of sample degradation was detected.

*Relative telomere length qPCR method*

In order to measure mean immune cell telomeres in the European badger we used a quantitative PCR (qPCR) approach. This assay has advantages and disadvantages over other methods available which are reviewed comprehensively in [1–4] and are not discussed further. Briefly, two sets of primers are designed: one to target repeat sequences associated with telomeric regions (TTAGGG) and a second to a non-variable copy number control gene (we used inter-photoreceptor retinoid-binding protein (IRBP): Ascension number AB082979). Target sequences are amplified using realtime qPCR in the presence of either a fluorescent nonspecific intercalating dye (used here) or a sequence specific fluorescent reporter molecule. Relative telomere length is calculated as the ratio of fluorescence from the telomeric amplicon compared to that from the non-variable copy gene standardised to a common sample (‘gold sample’) run on all plates (see equations below).

*Primers*

During assay development seven primer pairs targeting four control genes were designed from *Meles meles* sequences available in the GenBank database: Inter-photoreceptor retinoid-binding protein (IRBP), Actin alpha cardiac muscle protein (ACTC), Transthyretin protein (TTR) and cystic fibrosis transmembrane conductance regulator (CFTR). Primer pairs which showed non-specific amplification or primer-dimer formation were discarded. A primer pair targeting the IRBP region was chosen (see Table 1.) owing to its superior performance and previous use in telomere assays [5] and phylogenetic work [6] (owing to it being non-variable in copy number).

*Plate setup*

High-purity salt-free primers were synthesised by Eurofins (see Table 1.), diluted and stored at -20°C until use. Non-skirted 96-well Polypropylene qPCR plates were loaded manually and sealed with Mx3000P/Mx3005P Optical Strip Caps (Agilent). Control gene (IRBP) and telomere reactions were run on separate plates owing to differing optimal reaction temperatures (data not shown). Telomere and IRBP primer concentrations were optimised to 400nM and 200nM respectively. All reactions were run in triplicate (technical replicates) and averaged prior to analysis. In order to account for differences in amplification efficiencies between different plates, a standard curve was run on all plates comprising 1:2 serial dilutions of a pool of DNA from 10 individuals of unknown age (resulting in total DNA concentrations of 20, 10, 5, 2.5 and 1.25ng). In order to calculate a relative telomere length value for all samples, the 5ng dilution of the standard curve was used as the ‘golden sample’ to which all other samples were compared. See Table S2 for a schematic representation of the plate setup.

Individuals were randomly allocated to qPCR plates (all samples from a given individual were run on the same plate in order remove the impact of inter-plate variation on within-individual telomere length comparisons). The final reaction volume was 20ul containing 10ul of Brilliant II SYBR® Green Low ROX QPCR Master Mix (Agilent), 4ul nuclease free water (Fisher), 1ul each of forward and reverse primers (see Table 1) and 4ul of 1.25ng/ul DNA sample (or 4ul nuclease free water for the no template control). Reactions were run on the Stratagene Mx3000P qPCR system using a two-step reaction profile (Control Gene: 10 mins at 95°C, followed by 40 cycles of 30s at 95°C and 1min at 60°C, Telomere: 10 mins at 95°C, followed by 40 cycles of 30s at 95°C and 1min at 56°C). Fluorescence was recorded at the end of the low temperature annealing/extension step. LinRegPCR (v2013.0) was used to correct for baseline fluorescence and determine the window of linearity per amplicon. The threshold values (Nq) were set at the centre of the window in linearity for each amplicon (Nq = 0.22 and 0.17 (log fluorescence units) for IRBP and telomere reactions respectively). Threshold cycle values (Cq) for each sample were then determined as the cycle at which the amplification plot crossed the Nq. Primer specificity was confirmed through melt curve analysis (see Figure S2) and observation of a single band of the expected size after electrophoresis on a 3% agarose gel.

*Calculations*

Cq values were plotted against log concentration in order to determine the amplification efficiency of both IRBP and Telomere primers for each plate pair run. Across all plates, the mean standard curve amplification efficiencies were 99.9% (SE ± 1.5) for IRBP primers and 99.2% (SE ± 0.9) for the telomere primers. The R^2^ for each standard curve was >0.99.

First, two initial starting quantities (X_0_) were calculated for each sample, one from its telomere plate (X_0_ TEL) and one from its IRBP plate (X_0_ IRBP). To reconcile for amplification efficiency differences between plate runs we used the following equation:

X_0_ = 10^(Cq-b)/m^

Where Cq = Cycle at which the focal sample crosses the threshold (Nq), b = plate specific intercept of the log of the standard curve and m = plate specific slope of the log of the standard curve.

The amount of telomere in the focal sample was then normalised to the initial quantity of DNA in the sample by calculating:

X_0_ sample = X_0_ TEL / X_0_ IRBP

Finally, relative telomere length (RTL) was calculated by normalising the focal sample to the golden sample:

RTL = X_0_ sample / X_0_ golden sample

*Repeatability*

Amplicon specific within-plate variability was determined by examining the standard deviation of the triplicate Cq values for each sample across each plate. The median and inter-quartile range of the standard deviations across all samples (n=361) was 0.054 (0.036-0.082) for the IRBP primers and 0.097 (0.059- 0.14) for the telomere primers. In order to determine between-plate repeatability, 21 randomly selected samples (a single plate) were each run three times (each run once for telomere and once for IRBP, totalling 6 plates). The coefficient of variation in the relative telomere length estimates across all samples was 7.5%.

*Absolute Telomere Length Estimation*

The 21 samples selected for between-plate repeatability analysis (see above) were also subjected to the absolute telomere estimation method described by O’Callaghan & Fenech (2011). This method allows the calibration of relative telomere length estimates obtained using qPCR to known quantities of synthetic telomere and control gene oligomers (see Table 1 for sequences used here). In addition to the standard curve required for the relative method (see above) we included ten-fold dilutions of known concentrations of each synthetic oligomer on the same plate. Determining where each DNA sample crosses the synthetic standard curve for each amplicon can be used in order to determine the absolute quantity of telomere in each sample. Melt-curve analysis showed that primer products from both DNA and oligomer templates were specific (single peak) and had the same melt temperatures (Figure S1). As synthetic oligomers may have different amplification efficiencies to biologically extracted DNA (leading to bias in absolute telomere quantities), we calculated absolute starting quantities for each amplicon (A_0_: kb for telomere amplicon and diploid genomes for IRBP amplicon) whilst reconciling for differences in amplification efficiency between synthetic oligomers and extracted DNA samples as follows:

$$A_{0}= {E_{DNA}}^{b_{Oligo}logE_{DNA}\left( E_{Oligo} \right)-{Cq}_{sample}}$$

Where DNA = Biological extracted DNA, Oligo = synthetic oligomer, Cq = Cycle at which the focal sample crosses the threshold (Nq), b = intercept of the log of the standard curve, E = Efficiency of standard curve 10^(1/-m)^ and m = slope of the log of the standard curve.

We then standardised the absolute amount of telomere to the number of diploid genomes contained in the sample as follows:

Absolute TL = A_0_ TEL / A_0_ IRBP

*Relative to Absolute Conversion*

The estimates derived from the relative and absolute methods for these 21 samples were linearly related and highly correlated (R^2^ >0.99, Figure S2). Given that badgers have 22 chromosome pairs (44 chromosomes = 88 telomeres) and that the IRBP is a single copy gene [6], it was possible define an equation for the conversion of the relative telomere lengths into absolute telomere length per chromosome end:

Absolute TL Estimate (Kb) = (8.5 + 932.9*(RTL Estimate))/88

We estimated the average immune cell telomere length in our population of European badgers to be ~10kb, which is similar to immune cell telomere length estimates in humans (~15kb in young individuals [8]). It is important to note that the average immune cell telomere length estimate quoted here must be treated with caution. This estimate has not been validated a secondary direct methodology (such as TRF) [1,2].

**Figure S1.** Shows the melt curve analysis of both DNA and oligomer templates.


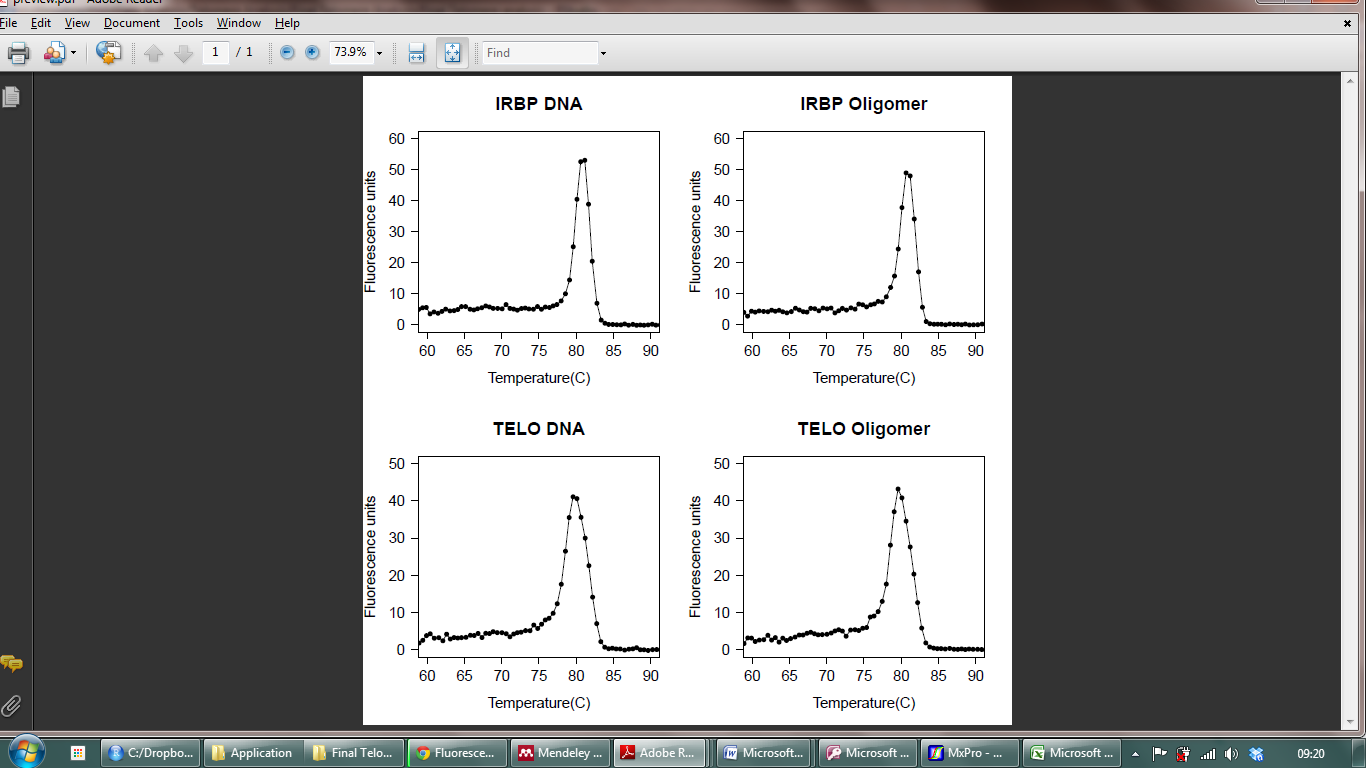


**Figure S2.** shows the correlation between absolute telomere length estimates and relative telomere length estimates for the 21 randomly selected samples.

­


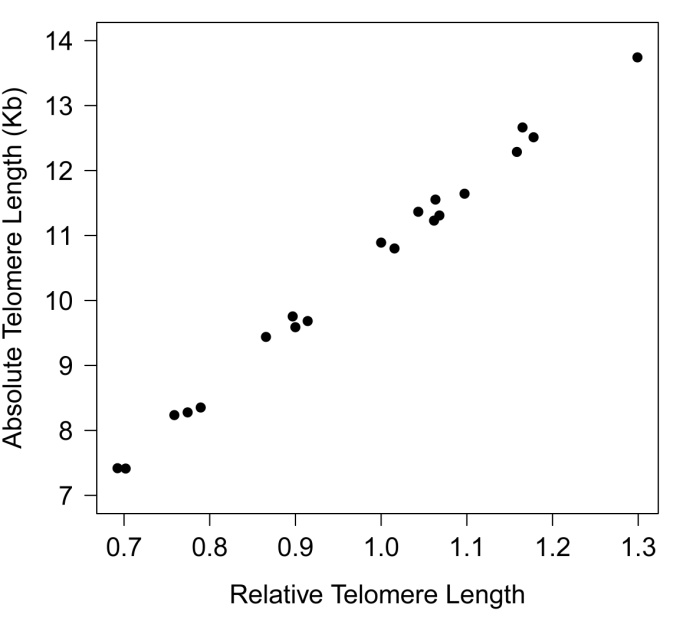


**Table S1.** Details the primer and oligonucleotide sequences used in the qPCR experiment.

| **Name** | **Target** | **Sequence** |
| --- | --- | --- |
|  |  |  |
| Tel1b | Telomeric Region | 5’-CGGTTTGTTTGGGTTTGGGTTTGGGTTTGGGTTTGGGTT-3’ |
| Tel2b | Telomeric Region | 5’-GGCTTGCCTTACCCTTACCCTTACCCTTACCCTTACCCT-3’ |
| IRBP-F | Inter-photoreceptor retinoid-binding protein (Ascension# AB082979) | 5’-GCCACATTTCTGGTATCCCCT-3’ |
| IRBP-R | Inter-photoreceptor retinoid-binding protein (Ascension# AB082979) | 5’-GGGCGGTCGTAGATGGTATC-3’ |
| Oligo-IRBP | NA | GCCACATTTCTGGTATCCCCTACTTCATCTCCTACCTGCACC  CAGGGAACACAGTCCTGCACGTGGATACCATCTACGACCG  CCC |
| Oligo-Telo | NA | (TTAGGG)_14_ |

**Table S2.** Shows a schematic of the standardised plate qPCR plate set-up. All numbers refer to the replicate of an individual sample, NTC = No Template Control, GS = Gold Sample, numbers preceded by S denote standard curve dilutions whereby: S1 = 20ng, S2 = 10ng, S3 = 5ng, S4 = 2.5ng, and S5 = 1.25ng. For the absolute qPCR telomere length estimation and inter-plate variation experiment, samples 3-7 were replaced by one in ten dilutions of known concentrations of synthesised oligomers (see table 1).

|  | **1** | **2** | **3** | **4** | **5** | **6** | **7** | **8** | **9** | **10** | **11** | **12** |
| --- | --- | --- | --- | --- | --- | --- | --- | --- | --- | --- | --- | --- |
| **A** | S1 | S1 | S1 | 3 | 3 | 3 | 11 | 11 | 11 | 19 | 19 | 19 |
| **B** | S2 | S2 | S2 | 4 | 4 | 4 | 12 | 12 | 12 | 20 | 20 | 20 |
| **C** | S3/GS | S3/GS | S3/GS | 5 | 5 | 5 | 13 | 13 | 13 | 21 | 21 | 21 |
| **D** | S4 | S4 | S4 | 6 | 6 | 6 | 14 | 14 | 14 | 22 | 22 | 22 |
| **E** | S5 | S5 | S5 | 7 | 7 | 7 | 15 | 15 | 15 | 23 | 23 | 23 |
| **F** | 1 | 1 | 1 | 8 | 8 | 8 | 16 | 16 | 16 | 24 | 24 | 24 |
| **G** | 2 | 2 | 2 | 9 | 9 | 9 | 17 | 17 | 17 | 25 | 25 | 25 |
| **H** | NTC | NTC | NTC | 10 | 10 | 10 | 18 | 18 | 18 | 26 | 26 | 26 |

**References**

1. Nussey DH, Baird D, Barrett E, Boner W, Fairlie J, et al. (2014) Measuring telomere length and telomere dynamics in evolutionary biology and ecology. Methods Ecol Evol 5: 299–310. Available: http://doi.wiley.com/10.1111/2041-210X.12161. Accessed 23 May 2014.

2. Foote CG, Vleck D, Vleck CM (2013) Extent and variability of interstitial telomeric sequences and their effects on estimates of telomere length. Mol Ecol Resour 13: 417–428. Available: http://www.ncbi.nlm.nih.gov/pubmed/23398661. Accessed 24 October 2013.

3. Aubert G, Hills M, Lansdorp P (2012) Telomere length measurement—Caveats and a critical assessment of the available technologies and tools. Mutat Res … 730: 59–67. Available: http://www.sciencedirect.com/science/article/pii/S0027510711000868. Accessed 31 August 2014.

4. Aviv A, Hunt SC, Lin J, Cao X, Kimura M, et al. (2011) Impartial comparative analysis of measurement of leukocyte telomere length/DNA content by Southern blots and qPCR. Nucleic Acids Res 39: e134. Available: http://www.pubmedcentral.nih.gov/articlerender.fcgi?artid=3203599&tool=pmcentrez&rendertype=abstract. Accessed 31 August 2014.

5. Izzo C, Hamer D, Bertozzi T, Donnellan S, Gillanders B (2011) Telomere length and age in pinnipeds: The endangered Australian sea lion as a case study. Mar Mammal Sci 27: 841–851. Available: http://doi.wiley.com/10.1111/j.1748-7692.2010.00450.x. Accessed 4 July 2014.

6. Sato JJ, Hosoda T, Wolsan M, Tsuchiya K, Yamamoto M, et al. (2003) Phylogenetic relationships and divergence times among mustelids (Mammalia: Carnivora) based on nucleotide sequences of the nuclear interphotoreceptor retinoid binding protein and mitochondrial cytochrome b genes. Zoolog Sci 20: 243–264. Available: http://www.ncbi.nlm.nih.gov/pubmed/12655187.

7. O’Callaghan NJ, Fenech M (2011) A quantitative PCR method for measuring absolute telomere length. Biol Proced Online 13: 3. Available: http://www.pubmedcentral.nih.gov/articlerender.fcgi?artid=3047434&tool=pmcentrez&rendertype=abstract. Accessed 1 March 2012.

8. Vera E, Bernardes de Jesus B, Foronda M, Flores JM, Blasco MA (2012) The rate of increase of short telomeres predicts longevity in mammals. Cell Rep 2: 1–6. Available: http://www.ncbi.nlm.nih.gov/pubmed/23022483. Accessed 8 October 2012.

9. Richards S, Whittingham M, Stephens P (2011) Model selection and model averaging in behavioural ecology: the utility of the IT-AIC framework. Behav Ecol Sociobiol 65: 77–89. Available: http://www.springerlink.com/index/10.1007/s00265-010-1035-8. Accessed 22 March 2013.
