# Supplementary material for: Age-Related Declines and Disease-Associated Variation in Immune Cell Telomere Length in a Wild Mammal
Source: PLoS One. 2014 Sep 30;9(9):e108964. doi: 10.1371/journal.pone.0108964 (PMC4182606; doi:10.1371/journal.pone.0108964)
Supplement: Information S2 — Full model selection table output from data analysis. Where: numbers denote β-estimates of continuous variables and ‘X’ denotes categorical variables included in each given model (row), INT = Intercept, MA = Mean Age (Years), DA = Delta Age (Years), SMI = Scaled Mass Index, TB = bTB infection status, : = interactions terms, df = degrees of freedom, LogLik = Log Likelihood, Δ = change in AICc from best model, W = Model Weight, AW = Strength of support for top model set (Δ AIC<6 from the best supported model and removal of more complex models with less support than a simpler nested version [9]), and bold typeface denotes the models in the top model set. (DOCX) [file pone.0108964.s003.docx]

**Information S2: Full Model Selection Output**

| INT | MA | DA | MA^2^ | DA^2^ | DA:MA | SEX | SMI | TB | DA:SEX | DA:SMI | DA:TB | MA:SEX | MA:SMI | MA:TB | SEX:SMI | SEX:TB | df | logLik | AICc | ΔAICc | W | AW |
| --- | --- | --- | --- | --- | --- | --- | --- | --- | --- | --- | --- | --- | --- | --- | --- | --- | --- | --- | --- | --- | --- | --- |
|  |  |  |  |  |  |  |  |  |  |  |  |  |  |  |  |  |  |  |  |  |  |  |
| **10.01** | **-0.14** | **-0.47** |  |  |  |  |  | **X** |  |  |  |  |  |  |  |  | **9** | **-615** | **1249.3** | **0.00** | **0.19** | **0.7** |
| 10.05 | -0.13 | -0.45 |  | -0.60 |  |  |  | X |  |  |  |  |  |  |  |  | 10 | -615 | 1249.9 | 0.67 | 0.13 | 0.0 |
| **10.13** | **-0.13** | **-0.46** |  |  |  |  |  |  |  |  |  |  |  |  |  |  | **7** | **-618** | **1251.0** | **1.70** | **0.08** | **0.3** |
| 10.00 | -0.13 | -0.47 | 0.00 |  |  |  |  | X |  |  |  |  |  |  |  |  | 10 | -615 | 1251.4 | 2.11 | 0.07 | 0.0 |
| 10.17 | -0.12 | -0.44 |  | -0.60 |  |  |  |  |  |  |  |  |  |  |  |  | 8 | -618 | 1251.6 | 2.31 | 0.06 | 0.0 |
| 10.02 | -0.09 | -0.45 | -0.01 | -0.63 |  |  |  | X |  |  |  |  |  |  |  |  | 11 | -615 | 1252.0 | 2.69 | 0.05 | 0.0 |
| 10.13 | -0.13 | -0.69 |  |  | 0.07 |  |  |  |  |  |  |  |  |  |  |  | 8 | -618 | 1252.3 | 3.00 | 0.04 | 0.0 |
| 10.06 | -0.13 | -0.46 |  |  |  | X |  |  |  |  |  |  |  |  |  |  | 8 | -618 | 1252.4 | 3.15 | 0.04 | 0.0 |
| 10.13 | -0.13 | -0.48 |  |  |  |  | 0.04 |  |  |  |  |  |  |  |  |  | 8 | -618 | 1252.8 | 3.58 | 0.03 | 0.0 |
| 10.09 | -0.12 | -0.44 |  | -0.65 |  | X |  |  |  |  |  |  |  |  |  |  | 9 | -617 | 1252.9 | 3.58 | 0.03 | 0.0 |
| 10.06 | -0.13 | -0.72 |  |  |  | X |  |  | X |  |  |  |  |  |  |  | 9 | -617 | 1252.9 | 3.68 | 0.03 | 0.0 |
| 10.09 | -0.09 | -0.46 | 0.00 |  |  |  |  |  |  |  |  |  |  |  |  |  | 8 | -618 | 1253.0 | 3.70 | 0.03 | 0.0 |
| 9.97 | -0.11 | -0.47 |  |  |  |  |  | X |  |  |  |  |  | X |  |  | 11 | -615 | 1253.3 | 4.05 | 0.03 | 0.0 |
| 10.01 | -0.14 | -0.55 |  |  |  |  |  | X |  |  | X |  |  |  |  |  | 11 | -615 | 1253.3 | 4.07 | 0.02 | 0.0 |
| 10.17 | -0.12 | -0.47 |  | -0.62 |  |  | 0.05 |  |  |  |  |  |  |  |  |  | 9 | -617 | 1253.4 | 4.14 | 0.02 | 0.0 |
| 10.11 | -0.06 | -0.44 | -0.01 | -0.65 |  |  |  |  |  |  |  |  |  |  |  |  | 9 | -617 | 1253.4 | 4.16 | 0.02 | 0.0 |
| 9.92 | -0.14 | -0.46 |  |  |  | X |  | X |  |  |  |  |  |  |  | X | 12 | -614 | 1253.6 | 4.34 | 0.02 | 0.0 |
| 10.13 | -0.13 | -0.50 |  |  |  |  | 0.03 |  |  | 0.17 |  |  |  |  |  |  | 9 | -618 | 1254.2 | 4.92 | 0.02 | 0.0 |
| 10.02 | -0.11 | -0.46 |  |  |  | X |  |  |  |  |  | X |  |  |  |  | 9 | -618 | 1254.3 | 5.04 | 0.02 | 0.0 |
| 10.03 | -0.09 | -0.46 | 0.00 |  |  | X |  |  |  |  |  |  |  |  |  |  | 9 | -618 | 1254.4 | 5.17 | 0.01 | 0.0 |
| 10.13 | -0.13 | -0.49 |  |  |  |  | -0.03 |  |  |  |  |  | 0.02 |  |  |  | 9 | -618 | 1254.5 | 5.23 | 0.01 | 0.0 |
| 10.03 | -0.06 | -0.44 | -0.01 | -0.69 |  | X |  |  |  |  |  |  |  |  |  |  | 10 | -617 | 1254.7 | 5.42 | 0.01 | 0.0 |
| 10.09 | -0.09 | -0.48 | -0.01 |  |  |  | 0.04 |  |  |  |  |  |  |  |  |  | 9 | -618 | 1254.8 | 5.57 | 0.01 | 0.0 |
| 10.11 | -0.05 | -0.47 | -0.01 | -0.67 |  |  | 0.05 |  |  |  |  |  |  |  |  |  | 10 | -617 | 1255.2 | 5.93 | 0.01 | 0.0 |
| 10.06 | -0.13 | -0.51 |  |  |  | X | -0.06 |  |  |  |  |  |  |  | X |  | 10 | -617 | 1255.5 | 6.21 | 0.01 | 0.0 |
| 10.12 | -0.12 |  |  |  |  |  |  |  |  |  |  |  |  |  |  |  | 6 | -622 | 1256.0 | 6.74 | 0.01 | 0.0 |
| 9.79 |  | -0.45 |  |  |  |  |  |  |  |  |  |  |  |  |  |  | 6 | -623 | 1258.6 | 9.34 | 0.00 | 0.0 |
| 9.80 |  |  |  |  |  |  |  |  |  |  |  |  |  |  |  |  | 5 | -627 | 1263.4 | 14.09 | 0.00 | 0.0 |
